# Generalizing an outbreak cluster detection method for two groups: an application to rabies

**DOI:** 10.1098/rsos.250821

**Published:** 2025-11-12

**Authors:** Sarah Hayes, Kennedy Lushasi, Joel Changalucha, Lwitiko Sikana, Katie Hampson, Christl A. Donnelly, Pierre Nouvellet

**Affiliations:** ^1^ Institute of Infection, Veterinary and Ecological Sciences, University of Liverpool, Liverpool, UK; ^2^ Department of Statistics, University of Oxford, Oxford, UK; ^3^ Pandemic Sciences Institute, University of Oxford, Oxford, UK; ^4^ Environmental Health and Ecological Sciences Department, Ifakara Health Institute, Ifakara, United Republic of Tanzania; ^5^ Boyd Orr Centre for Population & Ecosystem Health, School of Biodiversity, One Health & Veterinary Medicine, University of Glasgow, Glasgow, UK; ^6^ School of Public Health, Imperial College London, London, UK; ^7^ Department of Ecology and Evolution, University of Sussex, Brighton, UK

**Keywords:** rabies, cluster detection, infectious disease outbreaks

## Abstract

Identifying linked cases of an infectious disease can improve our understanding of its epidemiology by distinguishing sustained local transmission from frequent introductions with little onward transmission. This evidence can, in turn, inform decisions on interventions. Knowledge of epidemiological distributions and reporting probabilities is key in identifying linked cases. However, with multi-host pathogens quantitative differences between hosts may need consideration. In this study, an existing graph-based approach to detecting outbreak clusters was extended to allow for group-specific reporting probabilities and epidemiological distributions and to assess the level and importance of assortative mixing. This method was applied to data on animal rabies cases in Tanzania. Group-specific differences in reporting probabilities and epidemiological distributions and the level of assortative mixing had a marked impact on the size and composition of clusters. Results of the rabies cases analysis supported higher reporting probabilities in domestic animals than wildlife, no difference in mean transmission distance between groups, and frequent inter-species transmission. The method described here could be applied to other multi-host or multi-group systems in which heterogeneities in reporting probabilities, distributional parameters and/or levels of mixing exist between groups. This would allow more accurate characterization of transmission dynamics and thus facilitate implementation of more effective interventions.

## 1. Introduction

The life-history of a pathogen can vary between affected groups, potentially obscuring the transmission dynamics and hindering the design of effective interventions (table 1). While identifying linked cases can clarify transmission pathways, existing methods rarely account for group-specific differences in epidemiological characteristics. In this study, we introduce a new method for identifying clusters of linked cases in scenarios involving two groups with distinct epidemiological characteristics. In this context, groups could relate to, for example, different species, age-classes or behaviours relevant to transmission. We demonstrate how this method can be used to understand the transmission dynamics of a multi-host pathogen circulating across different species.

**Table 1.** Glossary of terms.

assortative mixing	individuals with similar characteristics are more likely to be linked than those with differing characteristics [1]
clusters	an aggregation of disease cases that are grouped together in space or time. Throughout this paper we use the term in the context of groups of linked cases where transmission has occurred between individuals in that group either directly or indirectly due to missing links in the chain of transmission.
edges	the connections between individuals within the network [2,3]
graph pruning	removal of certain *edges* in the network graph; in the context of this paper, pruning refers to removing *edges* considered less likely to represent disease transmission events between *nodes* [2]
nodes	individuals within the network
serial interval	the time interval between the onset of clinical signs in a primary case and the onset of clinical signs in a secondary case infected by the primary case
spillover	transmission between different species
transmission trees	used to illustrate who-infected-whom by visualizing transmission between cases using directed networks, where *nodes* represent individual cases and *edges* represent transmission between those cases [4,5]

Reconstruction of transmission trees to establish who-infected-whom can provide detailed insights into the transmission dynamics of an infectious disease and inform decisions on interventions [6–9]. However, transmission tree reconstruction requires detailed epidemiological data and dense sampling to minimize missing data, and may also be computationally expensive. Depending on the research question, full reconstruction of transmission trees may not always be necessary and useful information can be gained through identification of clusters of linked cases even if transmission events within a cluster are not fully characterized [10–12]. Throughout this document we use the term ‘clusters’ to describe groups of cases that are linked together in space and time, probably through transmission, even though some of the cases involved may not be observed. This situation may be more likely to occur in animal diseases where detailed epidemiological data are more challenging to collect, but could also depend on the severity of clinical signs.

Identifying linked cases can improve our understanding of the epidemiology of an infectious disease by identifying whether sustained local transmission is occurring or whether cases are due to frequent introductions with little ongoing transmission, two situations which may require different control strategies [13,14]. With zoonotic infections, for example, identification of linked cases can be useful in understanding whether transmission is occurring from frequent spillover or due to the occurrence of human-to-human transmission [15]. Within human healthcare settings, identification of linked cases can help establish whether outbreaks are due to nosocomial transmission or repeated introductions from the community, thus enabling appropriate control strategies to be implemented [16,17].

When identifying clusters of linked cases of an infectious disease, spatio-temporal case data may be combined with information regarding the underlying epidemiology of the disease to determine whether cases are close enough in space and/or time to be feasibly linked. The distribution of serial intervals and/or distances between infected cases may be used to determine a cut-off value above which cases are deemed too far apart in time and/or space to be directly linked. (In some cases genetic data may allow the linkage of cases that would otherwise be considered too far apart in space and/or time to be linked [8,18].) If there are unobserved cases in the transmission chain between linked cases, this cut-off value will be higher. Accounting for under-reporting within data when linking cases is therefore essential. For pathogens circulating within a single species (e.g. human infectious diseases), epidemiological distributions and reporting probabilities may differ by age group, for example, while for multi-host pathogens (pathogens capable of infecting multiple species) epidemiological distributions and reporting probabilities may differ between species. Reporting probabilities within the different hosts may also vary, especially with infections involving both wildlife and domestic hosts. We could not find any existing methods for identification of clusters of linked cases which are able to incorporate these heterogeneities.

Cori *et al.* [19] proposed a graph-based method for cluster detection that incorporates multiple data sources (temporal, spatial and genetic data) and that specifically accounts for under-reporting. This published method assumes a single population and uses a single epidemiological distribution for each data source. We have extended this approach for multi-host network reconstruction accommodating differences in disease epidemiology across distinct host populations. The extension presented here can account for differing reporting probabilities and differing epidemiological distributions between two host populations (such as different age groups or different species). We apply this novel framework to rabies, a multi-host pathogen, capable of infecting any mammal.

Rabies is a zoonotic viral disease that causes tens of thousands of human deaths each year, the majority of these occurring in Africa (36.4%) and Asia (59.6%) [20]. Under-reporting of human rabies cases is a common occurrence in many areas where the disease is endemic and can hamper our ability to understand the local transmission dynamics and impact of this disease [21–25]. Given the extent of under-reporting of human rabies deaths, it is reasonable to assume that the level of reporting of animal rabies cases is also very low, although data on reporting probabilities in animals are scarce [26,27]. As well as differences in reporting probabilities, differences in other epidemiological parameters may exist between wildlife and domestic animals. For example, the home-ranges of different species, and thus the distance over which we may expect transmission to occur, may vary considerably. For dogs and jackals (two species of importance in rabies transmission), studies suggest that domestic dog home ranges are quite small with estimates frequently less than 0.1 km^2^ [28–30], even in areas where dogs are largely free-roaming. By contrast, the home ranges of jackal species are typically reported to be 10–20 km^2^, and in some cases home ranges of almost 65 km^2^ are reported [31–33].

In this study, we present our framework for network reconstruction and apply it to rabies cases in domestic animals and wildlife in south-east Tanzania and explore how differences in epidemiological distributions and reporting probabilities can affect the size and characteristics of identified clusters. Rabies lends itself well to this framework as it is a directly-transmitted multi-host pathogen. In the south-east Tanzania study area, two hosts (dogs and jackals) are responsible for the vast majority of animal rabies cases and domestic dogs are frequently free-roaming [34–36] facilitating inter-species mixing.

## 2. Methods

A brief overview of the published method is first provided, followed by a description of our extension. An application of the framework to animal rabies case data from south-east Tanzania is then described.

### 2.1. Published method for cluster detection

A graph-based approach to cluster detection that combines multiple data sources is described in full by Cori *et al*. [19]. In brief, for each data source (spatial and temporal data in this study) pairwise distances are calculated for all cases and used to create graphs in which nodes represent the cases. We use the term ‘distance’ in a multivariate sense to describe both spatial and temporal distance. The edges between the cases (nodes) are labelled with the pairwise distances. Graphs are ‘pruned’ so that only those edges which have a pairwise distance below a specified cut-off value are retained.

The separate graphs for each distance variable are then merged by intersection to produce a single graph containing only those edges present in all data sources.

The cut-off value used for pruning is key to identifying clusters and has a marked effect on the resulting clusters. This cut-off value is determined by combining epidemiological information (e.g. serial interval distribution and/or distance kernel distribution) and case reporting probability, to produce a probability density function (PDF) for the distance between two cases, having accounted for under-reporting. The cut-off value is determined from the PDF using a user-selected percentile (e.g. 95th percentile).

In the published method [19], estimation of the PDF is performed within an analytical framework and implemented within an R package called ‘vimes’.

### 2.2. Novel extension to incorporate two groups with differing parameters

In this extension to the published method, we allow for transmission between two groups with differing epidemiological distributions and/or reporting probabilities. The presence of two groups allows for multiple transmission types, specifically, between two individuals from group 1 (G1–G1 (the convention used throughout is that the first listed is the primary case)), between two individuals in group 2 (G2–G2) and inter-group or ‘mixed’ transmission (G1–G2 or G2–G1). We do not discriminate between the two types of inter-group transmission and class both as ‘mixed’.

A simulation approach is implemented to produce the PDFs used to determine the cut-off values required for graph pruning. Simulation is used as it was considered the most efficient way to incorporate group-specific parameters. The user specifies the mean and standard deviation of the transmission distance for each group and a choice of parametric distribution for each type of data, and the reporting probability for each group. For each data type, a non-branching chain of individuals is simulated with transmission considered to pass along this chain in one direction. The user defines the number of individuals to be used within the simulation, but it is recommended to simulate at least 1 000 000 individuals as larger numbers provide more consistent results. Distances between individuals within the simulation are generated using the summary statistics and parametric distribution specified for the particular data type. Individuals within the simulation are marked as ‘observed’ or ‘unobserved’ based on the reporting probability. Distances between observed individuals are then extracted to produce the PDFs.

The simulation method was initially applied to a single group as a validation (i.e. to enable checks for consistency with the published method). Full details of the simulation method and checks undertaken are presented in electronic supplementary material (electronic supplementary material, Methods—Single group simulation).

The simulation method was then extended to incorporate two groups. The proportion of each group to be used within the simulation is calculated based on the number of cases in each group reported in the data being analysed and the assumed reporting probability for each group, both of which are entered by the user.

The total number of cases (observed and unobserved) that occurred in each group is estimated based on the number of observed cases and the reporting probability for that group,


(2.1) $$\left[{\hat{N}}_{i}\right]=\frac{O_{i}}{\rho_{i}}$$

where ${\hat{N}}_{i}$ is the estimated total number of cases in group *i* (observed and unobserved)*, O_i_* is the number of observed cases in group *i* and $\rho_{i}$ is the reporting probability for group *i*.

The proportion of each group to be used in the simulation is thus

(2.2) $$\pi_{i}=\frac{\left[{\hat{N}}_{i}\right]}{\sum_{i=1}^{g}[{\hat{N}}_{i}]}$$

where *π_i_* is the proportion of group *i* to include in the simulation and *g* is the number of groups.

**Figure 1.**
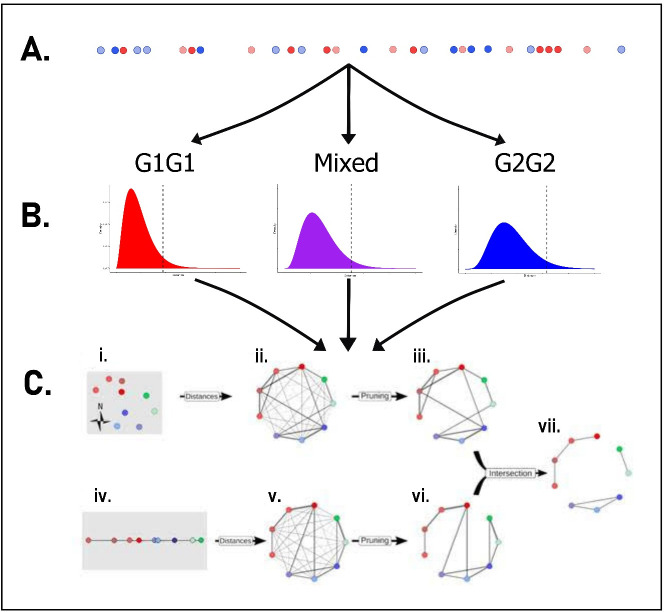
Schematic illustration of the extension of the graph-based method for cluster detection incorporating two groups. Panels (A) and (B) illustrate the process of deriving the cut-off values using simulated data, while panel (C) depicts the application of these cut-off values to the observed data being analysed. (A) For each data stream, a non-branching chain of transmission is generated containing simulated individuals from each group. The reporting probability for each group from the observed data to be analysed is used to simulate whether the individual is observed or not. Red = simulated observed group 1 (G1) cases, pale red = simulated unobserved G1 cases, blue = simulated observed group 2 (G2) cases, pale blue = simulated unobserved G2 cases. (B) The distances between the observed cases are extracted for each of the transmission types and used to create probability density functions (PDFs) for each transmission type. Cut-off values are extracted from these PDFs using a user-defined percentile (95th percentile shown by dotted lines). (C) Cut-off values generated for each simulated data stream are used for graph pruning for the observed case data. In this example, two data streams are considered: the spatial locations of the cases (i) and the dates of these cases (iv). The three ‘actual’ outbreak clusters are identified in red, blue and green, using different shadings to identify individual cases. Each data source defines a fully connected graph where nodes represent cases and edges are weighted by the spatial (ii) and temporal distances (v). Thicker edges represent smaller weights (distances) between cases. Each graph is pruned separately, removing edges whose weight exceeds the cut-off for that type of transmission. (iii, vi). The intersection of these graphs defines a new graph which retains only edges present in every pruned graph (vii). The resulting clusters of cases indicate likely outbreak clusters. Panel (C) is modified from [19].

A non-branching chain of the user-specified number of individuals (recommended minimum 1 000 000) with the required proportion from each group is simulated. (We have demonstrated 1 000 000 simulated individuals works well for reporting probabilities down to 0.1, but for lower reporting probabilities the number may need to be increased as lower reporting probabilities will equate to a smaller number of observed cases.) A transmission type is recorded for each simulated individual reflecting transmission between that individual and the next individual in the simulation. In the two-group application, the user is required to specify a parametric distribution for the between-individual distances (e.g. temporal or spatial) and to enter summary statistics for the distances for each of the three transmission types. The summary statistics may be the same or may vary by transmission type. Distances between simulated individuals are generated based on these user-specified values. Individuals are assigned as observed or unobserved based on the reporting probability for each group and distances between observed cases are calculated. In addition to the distances between observed individuals, the type of transmission that is observed is also extracted. Distances between observed individuals are used to estimate a PDF for each transmission type, which is then used to generate cut-off values for each transmission type (illustrated in table 2 and figure 1). The proportion of the observed transmissions that are of each transmission type is also extracted from the simulation. Extensive checks were undertaken to ensure consistency with the published method (electronic supplementary material, Methods—Two groups simulation).

**Table 2.** Illustration of the simulation method for estimation of the serial interval probability density function with two groups. Example of first nine individuals simulated during estimation of the probability density function for the serial interval with two groups and missing cases. In this example, there are 313 and 236 individuals in groups 1 and 2 respectively, the reporting probability is 0.6 for both groups. The serial interval is assumed to follow a gamma distribution, characterized by its shape and scale (these parameters are estimated using the user-entered summary statistics). The parameters for the G1–G1 and the mixed transmissions are the same, but the G2–G2 transmissions come from a distribution with a larger mean (for illustration of the method). Observed individuals are shown in italics. See electronic supplementary material, table S1 for equivalent table illustrating the sampling of the spatial distance distributions.

simulation number	group	observed	transmission type	shape parameter	scale parameter	distance (days)	cumulative distance (days)	observed transmission type^a^	observed distance (days)^a^
1	G2	FALSE	G2–G1	0.570	48.833	76.2	0	—	—
*2*	*G1*	*TRUE*	*G1–G1*	*0.570*	*48.833*	*42.5*	*76.2*	*G1–G1*	*42.5*
*3*	*G1*	*TRUE*	*G1–G1*	*0.570*	*48.833*	*34.0*	*118.7*	*G1–G1*	*41.5*
4	G1	FALSE	G1–G1	0.570	48.833	7.5	152.7	—	—
*5*	*G1*	*TRUE*	*G1–G2*	*0.570*	*48.833*	*80.0*	*160.2*	*G1–G2*	*121.3*
6	G2	FALSE	G2–G2	0.023	6104.1	41.3	240.2	—	—
*7*	*G2*	*TRUE*	*G2–G1*	*0.570*	*48.833*	*0.4*	*281.5*	*G2–G1*	*0.4*
*8*	*G1*	*TRUE*	*G1–G2*	*0.570*	*48.833*	*15.4*	*281.9*	*G1–G2*	*15.4*
*9*	*G2*	*TRUE*	*—*	*—*	*—*	*—*	*297.3*	*—*	*—*

^a^ Observed transmission type and observed distance refer to the transmission type and distance between the animal in that row and the next observed animal.

Assortative mixing was incorporated within the method to allow for different levels of mixing between groups and within groups. Thus, contact may occur more frequently within groups than between groups, increasing the likelihood of within-group transmission. We refer to this parameter as the ‘assortativity parameter’, *θ*, which is defined by the user. When *θ* = 1, this represents random mixing irrespective of group. As the value of *θ* increases, more assortative mixing occurs. While a higher value of the assortativity parameter represents more assortative mixing, there is not a closed-form solution for assortativity. See electronic supplementary material (electronic supplementary material, Methods—Assortative mixing) for additional information. When the level of assortative mixing in unknown, the user may wish to enter a range of values for the assortativity parameter and assess the clusters produced to evaluate which values generate estimates most consistent with their data. This is illustrated in the application to rabies data detailed in the next section.

The effects of group-specific reporting probability, the assortativity parameter, and group-specific mean transmission distance on cut-off values and expected proportions were explored across a range of hypothetical scenarios with different combinations of parameters (table 3). (The mean transmission distance refers to the expected distance (temporal or spatial) between a (primary) infected case and a subsequent (secondary) case infected by that primary case.) For all of these combinations, the size of group 1 was set at 500 and group 2 at 300 and assortativity values ranging from 1 to 10 in increments of 0.1 were implemented. The cut-off values and expected proportions for each transmission type were recorded.

**Table 3.** Hypothetical scenarios used to evaluate the impact of group-specific values for reporting probability and mean transmission kernel, and of assortative mixing on cut-off values. For each of the hypothetical scenarios (A–L) assortativity parameters ranging from 1 to 10 in increments of 0.1 were evaluated.

hypothetical scenario label	reporting probability G1	reporting probability G2	mean transmission distance (standard deviation)		
			G1G1	mixed transmission	G2G2
A	1.0	1.0	30 (20)	30 (20)	30 (20)
B	1.0	1.0	30 (20)	30 (20)	30 (20)
C	1.0	1.0	30 (20)	30 (20)	30 (20)
D	1.0	0.5	30 (20)	30 (20)	30 (20)
E	1.0	0.5	30 (20)	30 (20)	60 (40)
F	1.0	0.5	30 (20)	60 (40)	90 (30)
G	0.5	0.5	30 (20)	30 (20)	30 (20)
H	0.5	0.5	30 (20)	30 (20)	60 (40)
I	0.5	0.5	30 (20)	60 (40)	90 (30)
J	0.4	0.25	30 (20)	30 (20)	30 (20)
K	0.4	0.25	30 (20)	30 (20)	60 (40)
L	0.4	0.25	30 (20)	60 (40)	90 (30)

The pruning method that was implemented in the published method was extended to allow different cut-off values to be specified for the three transmission types (G1–G1, G2–G2 and mixed). These cut-off values can be generated by the simulation method described above, or can be manually entered by the user.

All analyses were performed using R v. 4.3.1 statistical software [37]. The code is implemented in a new R package called vimesMulti [38]. A vignette illustrating its use is also available [39]. The package was created in and is compatible with R v. 4.3.1.

### 2.3. Application to rabies data

**Figure 2.**
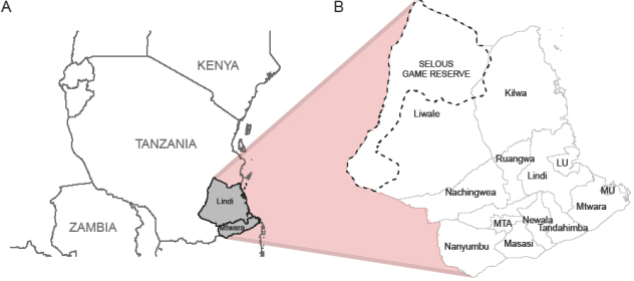
Maps of study area. (A) Tanzania showing location of study area (grey) and the regions of Lindi and Mtwara. (B) Districts within the two-region study area. In (A) Lindi and Mtwara refer to the regions of Lindi and Mtwara. In (B) Lindi and Mtwara refer to the districts of Lindi Rural and Mtwara Rural respectively. LU—Lindi Urban, MTA–Masasi Township Authority, MU—Mtwara Urban. The dotted line in (B) outlines the wildlife protected area of the Selous Game Reserve.

The method described above was applied to data on probable animal rabies cases that occurred between January 2011 and July 2019 within the 13 districts of Lindi and Mtwara regions of south-east Tanzania (figure 2), during a rabies elimination demonstration project delivered by the government of Tanzania, coordinated by the World Health Organization and funded by the Bill & Melinda Gates Foundation [40]. In total, 549 animal rabies cases were reported over the eight-and-a-half-year period. Of these 313 (57.0%) cases were in domestic animals (303 domestic dogs and 10 domestic cats) and 236 (43.0%) were in wildlife (221 jackals, 8 hyenas, 5 honey badgers and 2 leopards). Human victims of bites were used to identify probable rabid animals through reported details of the bite incident. This involved a mobile phone-based surveillance system that was used to record bite victims presenting to healthcare facilities requiring rabies post-exposure prophylaxis as described by Mtema *et al*. [41]. We extracted data from this system, validated it against paper-based records from health facilities across the study sites, and used it to initiate contact tracing. All bite victims and owners of biting animals were traced and interviewed to obtain details of each bite incident, as described in [42]. During interviews, the date and geographic coordinates of the bite incident and the species of biting animal were gathered. Details of the animal’s behaviour and the bite circumstances were used to assess whether the animal was considered likely to be rabid (based on the WHO definition of a probable animal rabies case [43]). Additional biting animals or bite victims identified during investigations were also traced and interviewed.

We assume the mean and standard deviation for the serial interval for rabies in animals to be 27.8 days and 36.9 days, respectively. For the distance kernel, we assume a mean distance between probable rabies cases in domestic dogs of 0.87 km with a standard deviation of 1.5 km. Data from a long-term contact tracing study in Serengeti district in northern Tanzania (described in [42,44]) were used for estimation of the summary statistics for these distributions. These data included the date and location of the bite incident for the primary rabid animals and the secondary cases that they infected, with information available for serial interval and distance kernel estimation in 1139 and 958 cases, respectively. The data from the Serengeti study were used as information regarding the serial interval and distance kernel for known transmission events within the south-east Tanzania data being analysed was limited and thus it was felt that the distributions would be better characterized using the larger Serengeti dataset (as described in [45]). Across Tanzania, domestic dogs are usually owned [36,46] and are predominantly kept outside. The number of unowned dogs is low. Across these rural settings they are almost all allowed to roam freely for at least part of the day [34–36]. Households with livestock are reportedly more likely to own dogs [47,48] .

The method described was used to detect clusters of linked cases in the south-east Tanzania animal rabies cases data. The data were divided into two groups with Group 1 (G1) comprising all cases in domestic animals and Group 2 (G2) comprising all wildlife cases. Cut-off values were generated using eight different parameter sets that were considered to represent plausible scenarios (outlined in table 4). Ten million individuals were used within the simulations and the 95th percentile was used to generate the cut-off values.

**Table 4.** Parameter sets used to generate cut-off values based on the animal rabies case data. The parameters for the serial interval distribution were kept constant across all scenarios.

scenario number	domestic animal (G1) reporting probability	wildlife (G2) reporting probability	G1–G1 mean of distance kernel (km)	G1–G2 and G2–G1 mean of distance kernel (km)	G2–G2 mean of distance kernel (km)
1	0.50	0.50	0.87	0.87	0.87
2	0.50	0.25	0.87	0.87	0.87
3	0.75	0.50	0.87	0.87	0.87
4	0.50	0.25	0.87	0.87	4.35
5	0.50	0.25	0.87	2.18	4.35
6	0.50	0.50	0.87	0.87	4.35
7	0.25	0.10	0.87	0.87	0.87
8	0.25	0.10	0.87	0.87	4.35

Data on the serial interval of rabies in species other than domestic dogs are sparse, thus the mean serial interval estimated for domestic dogs was used for all species and kept constant across all scenarios. The lognormal distribution was used for the serial interval as it had previously been found to be the best-fitting distribution for the animal rabies case data [45]. The reporting probability used for domestic animals was greater than or equal to that of wildlife with a reporting probability (*ρ*) of 0.5 deemed the most realistic estimate for domestic animal rabies cases in this part of Tanzania. Estimates of reporting probabilities for animal rabies cases using the same case detection methodology (contact tracing) across other areas of Tanzania are reported in the literature. For the Serengeti district of northern Tanzania, estimates of reporting probabilities range from 0.83 to 0.95 [49]. On Pemba Island reporting probabilities are estimated as 0.46–0.63 and 0.59–0.81 during an endemic period and an outbreak period respectively [50]. Here the reporting probability was assumed to be at the lower end of these estimates: contact tracing was less established in south-east Tanzania compared with Serengeti district and therefore more likely to reflect reporting probabilities similar to the earlier endemic period of implementation on Pemba Island rather than during the outbreak when tracing was more rapid.

Data on the distance kernel for rabies in wildlife are also lacking. Within the scenarios evaluated, the distance kernel for within-group transmission for wildlife was assumed to be greater than or equal to that of domestic animals. Where a larger value for wildlife was considered, a mean five times that of domestic animals was used to reflect the approximate difference in home ranges in domestic dogs and jackals described in the literature [28–33,51] with an intermediate distance of two-and-a-half times the mean used for mixed transmissions in one scenario (Scenario 5; see table 4). In addition to evaluating different values for the mean of the distance kernel, for each scenario two different parametric distributions (the Rayleigh distribution and the gamma distribution) were evaluated for the distance kernel. Cori *et al.* [19] noted that the Rayleigh distribution naturally emerges from measuring the distance travelled by a particle following two-dimensional Brownian motion. While not strictly embedded in a mechanistic explanation, the gamma distribution is frequently used in modelling due to its flexibility, and since it allows more/less dispersion surrounding the mean distance.

The effect of assortative mixing on the cut-off values generated within each of the scenarios was explored. In each scenario, simulations were run for values of the assortativity parameter *θ* ranging from 1.0 to 10.0 in 0.1 increments (91 values in total). The cut-off value and proportion of each transmission type were extracted from the simulations. The proportions of each transmission type were termed the ‘expected’ proportions. The cut-off values generated by the simulations for each transmission type were used for graph pruning.

We assessed which scenario and level of assortative transmission was most consistent with the data using the chi-squared goodness-of-fit test with one degree of freedom. Estimation was challenging as we assume missing cases in the data and so do not have access to the ‘true’ proportions of each transmission type for comparison with the ‘expected’ proportions generated by the simulations. As such, an approximation method based on the proportion of each transmission type within the identified clusters was used. Within the clusters identified following pruning, the proportion of each type of transmission between linked pairs was extracted. The method does not reconstruct transmission trees, it identifies groups of linked cases. As such, individuals within a cluster may be linked to multiple other individuals within the cluster and the number of linked pairs may exceed the number of transmissions that occurred. The proportion of each type of transmission that occurred between linked pairs within all clusters of two or more individuals identified by graph pruning was calculated. These were termed the ‘observed’ proportions of each transmission type. The expected proportions (from the simulation) and the observed proportions (from the processed data after pruning) were used to estimate the observed and expected number of each transmission type based on the number of individuals that were included within all clusters of two or more in clusters identified following graph pruning. A chi-squared test statistic was obtained for each of the 91 simulations with different values of *θ*. The assortativity parameter *θ* associated with the lowest chi-squared statistic (i.e. the best fit) was considered to indicate the degree of assortative mixing that best calibrated the cut-off values in the simulation to represent the data in each of the eight scenarios.

The number, size and composition of clusters obtained in the scenario that was most consistent with the data based on the chi-squared goodness-of-fit test are reported.

### 2.4. Sensitivity analyses

The date and location of the rabies cases exhibited uncertainty in 534 (97%) and 322 (59%) of cases, respectively. For the temporal data, the uncertainty was recorded as ±7 days (479 cases), ±14 days (9 cases) and ±28 days (46 cases). For the spatial data, the uncertainty was recorded as within 2 km (173 cases), 2–5 km (121 cases) and 5–10 km (28 cases). To explore the impact of this uncertainty on the results, 100 new datasets were generated from the existing data. In each of these datasets, for each case a new value for the date and location was uniformly sampled from within the window of uncertainty for that case. The cluster identification method was applied for each of the scenarios listed in table 4 as described for the original data above. The percentage of the 100 datasets where the chi-squared goodness-of-fit test was compatible with the data was recorded for each scenario.

## 3. Results

### 3.1. Assessment of the novel method

Results of the simulation using a single group were robust when compared with the published method for all scenarios explored when using 1 000 000 or more simulated individuals, with differences within the order of magnitude of the numerical error used for the published method (precision parameters). As the number of individuals used within the simulation increased there was an increase in the precision of the estimates for the cut-off values obtained and a decrease in the percentage difference between the cut-off values estimated by the simulation and those estimated by the published method. Detailed results of the comparisons are provided in electronic supplementary material (electronic supplementary material, Results—Single group simulation results).

The precision of the cut-off value estimates when using two groups was improved when the number of individuals in the simulation was increased and at higher reporting probabilities. As expected, the simulations using the different reporting probabilities but the same epidemiological distributions for two groups produced the same cut-off values as generated in the published method using the weighted mean reporting probability (within stochastic variation). Details of the results including the comparison of the serial interval cut-off values using the simulation method with two groups and different reporting probabilities and using a weighted mean in the published method are in electronic supplementary material (electronic supplementary material, Results—Two group simulation results and table S8).

Differences in group-specific values for reporting probabilities and mean transmission distance, and the value of the assortativity parameter all affected cut-off values (table 5). While values ranging from 1 to 10 were explored for the assortativity parameter, for values above 6, very little variation in the cut-off values generated was observed (suggesting that at this level mixing was largely assortative) so we give 6 as the upper limit in the table. When both reporting probabilities and means of the distributions were the same across all groups (table 5, A and G), cut-off values were the same for each transmission type. With perfect reporting, cut-off values were not affected by changes to the assortativity parameter, but differences in group-specific cut-off values were seen with differences in the mean transmission distances for the different groups (table 5, B and C). The biggest differences between group-specific cut-off values were seen when there were differences in both the group-specific reporting probabilities and the group-specific mean transmission distances and with higher levels of assortativity (table 5, E, F, K and L).

**Table 5.** Effect of changes in reporting probability, mean transmission distance and assortativity parameters on cut-off values and expected proportions in 12 hypothetical scenarios. Only 3 of the 10 assortativity parameters explored for each parameter set are shown as an illustration. Values generated using 500 individuals in G1 and 300 individuals in G2 and with 10 000 000 individuals within the simulation and using 95th percentile for the cut-off value.

hypothetical scenario	reporting prob. G1	reporting prob. G2	mean transmission distance (standard deviation)			trans. type	**cut-off values (using 95th percentile)**			expected proportions		
			G1G1	mixed trans.	G2G2		assortativity parameter			assortativity parameter		
							1	3	6	1	3	6
A	1.0	1.0	30 (20)	30 (20)	30 (20)	G1–G1	67.6	67.7	67.7	0.39	0.54	0.61
						mixed	67.7	67.5	67.6	0.47	0.17	0.02
						G2–G2	67.7	67.7	67.7	0.14	0.29	0.36
B	1.0	1.0	30 (20)	30 (20)	60 (20)	G1–G1	67.6	67.7	67.7	0.39	0.54	0.61
						mixed	67.7	67.5	67.6	0.47	0.17	0.02
						G2–G2	135	135	135	0.14	0.29	0.36
C	1.0	1.0	30 (20)	60 (40)	90 (40)	G1–G1	67.6	67.7	67.7	0.39	0.54	0.61
						mixed	135	135	135	0.47	0.17	0.02
						G2–G2	203	203	203	0.14	0.29	0.36
D	1.0	0.5	30 (20)	30 (20)	30 (20)	G1–G1	104	77.1	68.7	0.39	0.54	0.61
						mixed	104	114	118	0.47	0.17	0.02
						G2–G2	104	151	160	0.14	0.29	0.36
E	1.0	0.5	30 (20)	30 (20)	60 (40)	G1–G1	121	78.5	68.8	0.39	0.54	0.61
						mixed	160	183	192	0.47	0.17	0.02
						G2–G2	208	301	321	0.14	0.29	0.36
F	1.0	0.5	30 (20)	60 (40)	90 (30)	G1–G1	217	105	70.4	0.39	0.54	0.61
						mixed	262	296	308	0.47	0.17	0.02
						G2–G2	312	452	481	0.14	0.29	0.36
G	0.5	0.5	30 (20)	30 (20)	30 (20)	G1–G1	161	162	162	0.39	0.54	0.61
						mixed	161	162	162	0.47	0.17	0.02
						G2–G2	161	162	161	0.14	0.29	0.36
H	0.5	0.5	30 (20)	30 (20)	60 (40)	G1–G1	173	164	162	0.39	0.54	0.61
						mixed	193	208	210	0.47	0.17	0.02
						G2–G2	219	302	321	0.14	0.29	0.36
I	0.5	0.5	30 (20)	60 (40)	90 (30)	G1–G1	265	186	164	0.39	0.54	0.61
						mixed	307	327	329	0.47	0.17	0.02
						G2–G2	350	459	482	0.14	0.29	0.36
J	0.4	0.25	30 (20)	30 (20)	30 (20)	G1–G1	258	220	208	0.39	0.54	0.61
						mixed	257	270	278	0.47	0.17	0.02
						G2–G2	258	325	339	0.14	0.29	0.36
K	0.4	0.25	30 (20)	30 (20)	60 (40)	G1–G1	318	230	209	0.39	0.54	0.61
						mixed	341	402	433	0.47	0.17	0.02
						G2–G2	365	623	676	0.14	0.29	0.36
L	0.4	0.25	30 (20)	60 (40)	90 (30)	G1–G1	514	289	215	0.39	0.54	0.61
						mixed	549	635	678	0.47	0.17	0.02
						G2–G2	587	945	1015	0.14	0.29	0.36

Only the assortativity parameter influenced the expected proportions of each transmission type. The expected proportion of mixed transmissions decreased as the assortativity parameter increased while the expected proportions of within-species transmissions (G1–G1 and G2–G2) increased. The values for these proportions were the same for all reporting probabilities explored and for when the mean distance kernel was the same across all types of transmission or was higher for one group (table 5). There was little change in either the cut-off values or expected proportions for assortativity values above 6.0, reflecting little between-group mixing above this value.

### 3.2. Application to rabies data

Of the eight scenarios evaluated, three had chi-squared goodness-of-fit test statistics that corresponded to *p*-values greater than 0.05 with one degree of freedom (i.e. the models were consistent with the ‘observed’ data) with at least one of the assortativity parameters examined. The scenarios that were statistically consistent with the data were scenario 2 using the gamma distribution for the distance kernel and scenarios 2 and 7 using the Rayleigh distribution for the distance kernel. Of these three scenarios, scenario 2 using the gamma distribution for the distance kernel had the lowest chi-squared value (and correspondingly highest *p*‐value). All scenarios that were statistically consistent with the observed data were those where the distance kernel was the same for all transmission types and reporting probabilities were lower in wildlife (G2) compared with domestic animals (G1). Details of the scenarios, results for the chi-squared goodness-of-fit test and associated assortativity parameters are shown in table 6.

**Table 6.** Results from comparing the eight scenarios with the observed data. G1 comprised 313 observed cases and G2 comprised 236 observed cases with 10 000 000 individuals used within the simulation to obtain the cut-off values and expected proportions using the 95th percentile. Values for the assortativity parameter ranging between 1.0 and 10.0 at increments of 0.1 were explored for each scenario. The parameters for the serial interval distribution were constant across all scenarios. Scenarios with goodness-of-fit *p*-values >0.05 are shown in italics with the *p*-values shown in bold.

scenario	domestic animal (G1) reporting probability	wildlife (G2) reporting probability	G1–G1 mean distance kernel (km)	G1–G2 and G2–G1 mean distance kernel (km)	G2–G2 mean distance kernel (km)	gamma distribution		Rayleigh distribution	
						minimum chi-squared value (goodness-of-fit *p*‐value)	assortativity parameter with minimum chi-squared value (range of assortativity parameters with *p*‐value > 0.05 if applicable)	minimum chi-squared value (goodness-of-fit *p*‐value)	assortativity parameter with minimum chi-squared value (range of assortativity parameters with *p*‐value > 0.05 if applicable)
1	0.50	0.50	0.87	0.87	0.87	9.55 (0.002)	2.6	14.2 (<0.001)	3.5
*2*	*0.50*	*0.25*	*0.87*	*0.87*	*0.87*	*0.884* ***(0.347)***	*2.2* *(2.1–2.4)*	*2.34* ***(0.126)***	*3.1* *(2.9–3.4)*
3	0.75	0.50	0.87	0.87	0.87	5.30 (0.021)	3.0	10.5 (0.001)	3.5
4	0.50	0.25	0.87	0.87	4.35	743 (<0.001)	1.0	216 (<0.001)	1.0
5	0.50	0.25	0.87	2.175	4.35	545 (<0.001)	1.0	198 (<0.001)	1.3
6	0.50	0.50	0.87	0.87	4.35	448 (<0.001)	3.0	73.1 (<0.001)	2.5
*7*	*0.25*	*0.10*	*0.87*	*0.87*	*0.87*	39.0 (<0.001)	1.9	*1.76* ***(0.185)***	*2.4* *(2.3–2.6)*
8	0.25	0.10	0.87	0.87	4.35	828 (<0.001)	1.1	400 (<0.001)	1.0

We focus on the results from scenario 2 using the gamma distribution for the distance kernel as this were best supported by the data based on the chi-squared goodness-of-fit test (table 6). Results from the other two scenarios that were supported by the data (scenario 2 using the Rayleigh distribution as the distance kernel and scenario 7 using the Rayleigh distribution for the distance kernel) are reported in electronic supplementary material (electronic supplementary material, Results—Results from the two additional scenarios consistent with the data). Cut-off values and proportions for the best-fitting scenario are shown in table 7. The assortativity parameter for the best-fitting scenario was 2.2 suggesting that inclusion of assortative mixing was more likely to reflect the true situation than random mixing.

**Table 7.** Cut-off values and observed and expected proportions of transmissions associated with the best-fitting scenario. Reporting probabilities were 0.5 for domestic animals (G1) and 0.25 for wildlife (G2). The mean distance kernel was 0.87 km for all transmission types and the mean serial interval was 27.8 days. The gamma distribution was used for the distance kernel and the 95th percentile used for the cut-off value. The best-fitting assortativity parameter, *θ*, was 2.2.

transmission type	cut-off value for distance kernel using 95th percentile (km)	cut-off value for serial interval using 95th percentile (days)	proportion of transmission	
			expected^a^	observed^b^
G1–G1	5.92	212	0.42	0.41
mixed	6.55	262	0.29	0.28
G2–G2	7.14	313	0.28	0.30

^a^ Expected proportion refers to the proportion of each type of transmission extracted from the simulation.

^b^ Observed proportion refers to the proportion of the pairwise transmissions of each type between linked pairs in the clusters identified.

**Figure 3.**
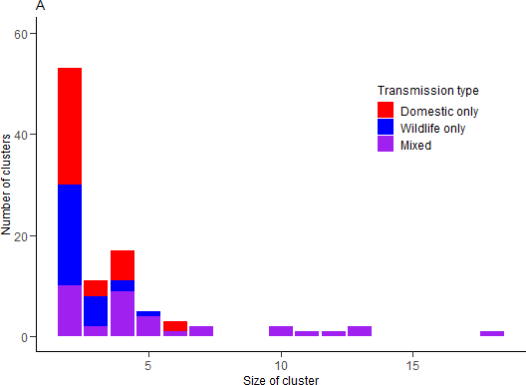
Cluster size and composition for best-fitting scenario. Reporting probabilities were 0.5 for domestic animals (G1) and 0.25 for wildlife (G2) and the mean distance kernel was 0.87 km for all transmission types. The best-fitting assortativity parameter was 2.2.

Of the 549 animals in the data, 351 (64%) were assigned to a cluster. Ninety-eight clusters of two or more animals were identified. Of the 98 identified clusters of two or more animals, 35 (36%) involved inter-species transmission. Inter-species transmission was present in all clusters of seven or more animals. Plots of the cluster size and composition are shown in figure 3.

**Figure 4.**
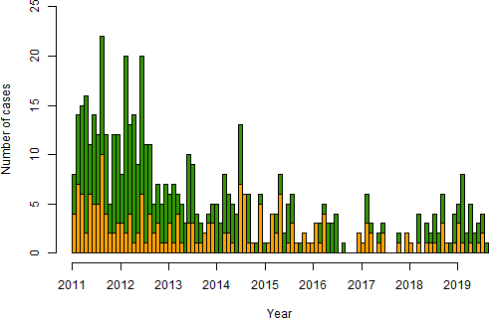
Number of animal rabies cases per 30-day period. The cases in green are assigned to a cluster, while those in orange are cases not assigned to a cluster.

**Figure 5.**
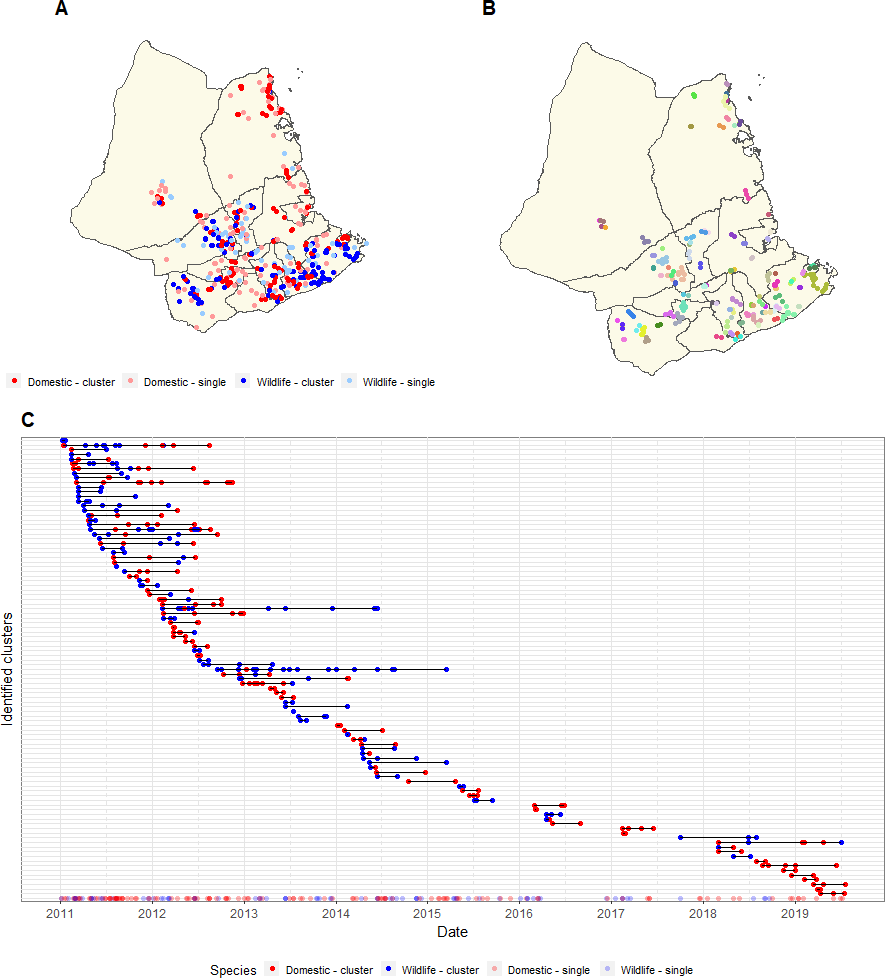
Location and timing of cases within clusters. (A) Map of study area with location of cases coloured by domestic or wildlife and whether or not they were assigned to a cluster. (B) Map showing cases assigned to clusters of three or more, coloured by cluster assignment. (C) Timeline showing timing of cases assigned to clusters of three or more coloured by domestic animals and wildlife. Only clusters of three or more are shown to aid visual interpretation The bottom row, shaded with lighter colours, are the cases not assigned to a cluster.

Of the 198 animals not attributed to a cluster, 124 (63%) were domestic animals and 74 (37%) were wildlife. (Within the complete dataset, 57% of animals are domestic animals and 43% are wildlife.) The temporal distribution of cases not assigned to a cluster is shown in figure 4. The locations of cases assigned to clusters of three or more and the timing of the cases assigned to these clusters is illustrated in figure 5.

### 3.3. Sensitivity analysis

Results of the sensitivity analysis provided further support for scenario 2 with the gamma distribution for the distance kernel being the best-fitting scenario to the data. Using the 100 datasets with resampled dates and locations drawn from the uncertainty windows for timing and location and assortativity parameters values of 2.2, 2.3 and 2.4, the percentage of the 100 datasets which were well fit based on the chi-squared goodness-of-fit was 84%, 96% and 90% respectively (i.e. very close to the 95% expectation).

Three additional scenarios also had results where at least one of the datasets was statistically consistent with the data. These were scenarios 2 and 7 using the Rayleigh distribution for the distance kernel and scenario 3 using the gamma distribution for the distance kernel. For these scenarios, the percentage of the 100 datasets that were well fit based on the chi-squared goodness-of-fit test were lower at 27% in scenario 2 (Rayleigh distribution), 20% in scenario 3 (gamma distribution) and 58% in scenario 7 (Rayleigh distribution). Details of the results can be found in electronic supplementary material (electronic supplementary material, Results—Sensitivity analysis).

## 4. Discussion

In this study, we extend a published method for outbreak cluster detection to allow for group-specific reporting probabilities and epidemiological distributions and highlight how influential these heterogeneities can be when identifying clusters of transmission. When this new method was applied to data on probable animal rabies cases from south-east Tanzania, the results suggested that the scenarios that were most compatible with the data involved higher reporting probabilities for rabies cases in domestic animals compared with wildlife, no difference between the mean transmission distance for infection for domestic animals and wildlife and support for moderate assortative mixing within wildlife and domestic animals. Inter-species transmission commonly occurred within the identified clusters.

Cut-off values are key in identification of linked cases. Our results show how differences in group-specific reporting probabilities and mean transmission distances impact these cut-off values. Where heterogeneities are expected to exist between groups, use of a cluster-identification method which can accommodate these heterogeneities, such as the one described here, would improve the accuracy of cluster identification and thus improve understanding of transmission dynamics. This would facilitate more targeted disease interventions. For example, if results indicated local disease transmission (e.g. by identification of large clusters), the focus might be on interrupting local transmission (e.g. through vaccination in those areas). Alternatively, if results suggested importation of cases from surrounding areas was more likely (due to identification of smaller clusters or singletons), a focus on preventing importations might be more appropriate. In the rabies example given here, if large clusters consisting solely of wildlife were identified, interventions targeting wildlife might be appropriate.

When the method was applied to rabies case data, eight scenarios were explored that were considered to reflect contrasting yet plausible estimates of the reporting probabilities and epidemiological distributions for the study area. While it would be possible to explore many more combinations of parameters, we focused on a justifiable subset given the context.

Estimates of reporting probabilities of animal rabies were derived from studies in other areas of Tanzania that used the same data collection method [49,50]. However, the proportion of wildlife rabies cases in this study is considerably higher than those other study areas. Contact tracing relies largely on reports from human victims of animal bites. Wildlife are presumed likely to have less contact with humans than domestic animals, which could conceivably lead to lower reporting probabilities. The scenarios identified as most compatible with the data were indeed those in which the reporting probability for cases in wildlife was lower than that for domestic animals. Reporting probabilities were assumed to be constant over the study period as the contact-tracing methodology was applied with consistent effort throughout the data collection period. The reported method does not accommodate temporal or spatial variations in reporting probabilities. Due to limited data for wildlife, the serial interval from domestic dogs was applied across all species, with future work needed to assess the impact of inter-species differences.

Specifying the mean of the distance kernel for wildlife as 4.35 km (five times that of domestic animals) was based on reports of home range sizes of domestic dogs and jackals and was considered relatively conservative as it is at the lower end of the home range sizes reported for jackals [31–33,51]. However, home range sizes may not accurately reflect mean transmission distances. The mean transmission distance of 0.87 km reported for domestic dogs in northern Tanzania is larger than many of the published estimates of domestic dog home range sizes [28–30]. One possible reason for this difference is that the reported transmission distances among domestic dogs include many ‘local transmissions’ (approximately 20% occur within the same household), with a small number of long-distance transmissions which may disproportionately affect the mean. Free-roaming domestic dog movements tend to be over-dispersed, with some travelling much further than the median recorded distances [28,30,52,53]. Other reasons for these long-distance transmissions could include human-mediated movement of dogs [54] or rabies-induced behavioural changes affecting movement patterns [49]. Social structure and seasonal changes in roaming behaviour due to mating, food availability and juvenile dispersal could all influence the transmission distance of rabies in wildlife. We have used the 95th percentile of the PDF for the distance kernel in this study. Higher percentile values could be used, but would increase the likelihood of linking cases that are not epidemiologically linked. The percentile value chosen should thus reflect the goal of the individual study.

The mean distance kernel for wildlife was the most challenging to estimate due to an absence of data. However, field studies to gather data on the distance kernel for rabies transmission would be challenging and raise ethical issues. While radio-collar studies in jackals have been performed to estimate home ranges, these may not reflect rabies transmission distances. The use of two-species compartmental models fitted to field data may be one option for obtaining estimates for this parameter.

Of the scenarios explored, those most compatible with the data were those that suggested some degree of assortative mixing. While there are limitations on these results due to uncertainty around the reporting probabilities and mean distance kernel for rabies transmission, this work illustrates that in the presence of two groups with differing reporting probabilities and epidemiological distributions, the degree of assortative mixing (and thus of assortative transmission) may have a substantial effect on the outcomes of outbreak cluster detection. Higher levels of mixing would be expected among conspecifics (as they are more likely to be present in the same areas and interact within family/social groups etc.), so a degree of assortative transmission is likely even if transmission occurs freely between species. All scenarios identified clusters containing both wildlife and domestic species and so while a degree of assortative transmission was supported, none suggested fully assortative transmission. These results suggest that rabies is unlikely to be maintained solely in species-specific cycles in the study area.

Of the 549 cases within the rabies case data, 198 were not assigned to a cluster. There are a number of possible reasons for this. For domestic dogs, some of these could result from human-mediated importations. A study from Bangui, a large city in the Central African Republic, suggested a rate of importation of approximately seven rabid dogs per year. While it is hard to directly compare the Bangui study with our study area it does suggest that human-mediated importations can occur commonly and may explain some of the unlinked domestic dog cases. The choice of the 95th percentile for generation of the cut-off values excludes a proportion of the transmissions that do occur over unusually long spatial and temporal transmission distances. The cases assigned to clusters are the observed cases and as we assume a high level of under-reporting for rabies (with reporting probabilities of 0.5 for domestic dogs and 0.25 for wildlife in the best-fitting scenario) it is possible that cases not assigned to a cluster are actually linked to unobserved cases.

The simulation method presented here showed good precision and accuracy in determining cut-off values when compared with the published method when 1 000 000 or more individuals were used within the simulation. As expected, increasing the number of individuals within the simulation improved the accuracy. The simulation method is fast enough to allow 10 000 000 individuals to be used routinely (18 s to run the simulation with two groups using 10  000 000 individuals for one value of the assortativity parameter on an i7 processor with 16.0 GB RAM). While the only distributions currently incorporated in the simulation method are the gamma or lognormal distribution for the serial interval and the Rayleigh and gamma distributions for the distance kernel, the simulation method could be easily extended to allow for more distance metrics and distributions. In addition, the same simulation framework could be used to extend the method to allow for more than two groups. The published method allows use of genetic data in cluster detection. A future extension of the simulation method presented (which was outside of the scope of this paper) could involve development of the method to incorporate genetic data, with assessment of necessary underlying assumptions (e.g. mutation rates) and subsequent validation using a dataset with temporal, spatial and genetic data available. (Genetic data were not available in the presented rabies cases data.)

In the simulation, to determine the cut-off values, a single chain of transmission is simulated in each instance. For endemic diseases, the assumption of one individual infecting, on average, one other individual is realistic (effective reproduction number (*R*_*t*_) ≈ 1). However, in an epidemic situation with high values of *R*_*t*_, where one individual may infect many others, the assumption of a single chain of transmission is less robust. This limitation is also applicable to the original published method.

Implementing assortative mixing within the simulation while keeping the desired proportions of each group and maintaining computational efficiency was challenging. The method that has been implemented is appropriate for exploring the impact of assortative mixing on possible outcomes rather than for measuring the absolute level of assortative mixing that is occurring. Another limitation of the analysis is the use of processed data for the ‘observed’ data and reliance on assumed reporting probabilities as fully observed transmission chains were not available. As such the results obtained serve as a guide to the degree of assortative mixing and are useful for comparing scenarios rather than providing definitive estimates of mixing.

## 5. Conclusions

A novel method of cluster detection is presented that allows for two groups with group-specific reporting probabilities and epidemiological distributions while also exploring the importance of assortative mixing. The method is most applicable to endemic diseases where the reproduction number is relatively low and where some information is available on the epidemiological distributions within the transmission system. It may be particularly useful for situations where full transmission tree reconstruction is not possible (e.g. due to difficulties in conducting detailed epidemiological investigations, such as in animal-to-animal transmission or resource-limited settings). The results highlight the importance of accurate estimation of distributional parameters and reporting probabilities in reliably identifying clusters. This method could be applied to other multi-host endemic disease scenarios such as the circulation of bovine tuberculosis among wildlife and domestic animals in south Africa [55,56]. As well as being applicable to multi-host pathogens, it could also be used for single-host pathogens where within-host groups (such as age-groups) exhibit differences in reporting probabilities, epidemiological distributions and/or assortative mixing. The results of applying this new method to data on probable animal rabies cases in south-east Tanzania suggest that between-species transmission and mixed-species clusters are common and that some assortative transmission is occurring.

## Data Availability

The R package vimesMulti is available at GitHub [38]. A vignette illustrating its use is also available [39]. The code for the analyses of the rabies data using the vimeMulti package are available at GitHub [57]. Data regarding the locations and timing of the rabies cases are available in the Dryad Digital Repository [58]. Supplementary material is available online [59].
